# Interactome Analysis of KIN (Kin17) Shows New Functions of This Protein

**DOI:** 10.3390/cimb43020056

**Published:** 2021-07-22

**Authors:** Vanessa Pinatto Gaspar, Anelise Cardoso Ramos, Philippe Cloutier, José Renato Pattaro Junior, Francisco Ferreira Duarte Junior, Annie Bouchard, Flavio Augusto Vicente Seixas, Benoit Coulombe, Maria Aparecida Fernandez

**Affiliations:** 1 Departamento de Biotecnologia, Genética e Biologia Celular, Universidade Estadual de Maringá, Av. Colombo, 5790, Maringá 87020-900, Brazil; vapigas@gmail.com (V.P.G.); anelise.andre@gmail.com (A.C.R.); junior.fduarte@gmail.com (F.F.D.J.); 2 Institut de Recherches Cliniques de Montréal, 110 Avenue des Pins Ouest, Montreal, QC H2W 1R7, Canada; phil.clou@gmail.com (P.C.); anniebouchard78@gmail.com (A.B.); benoit.coulombe@ircm.qc.ca (B.C.); 3 Departamento de Tecnologia, Campus Umuarama, Universidade Estadual de Maringá, Av. Ângelo Moreira da Fonseca, 1800, Umuarama 87506-370, Brazil; pattoze@gmail.com (J.R.P.J.); favseixas@gmail.com (F.A.V.S.)

**Keywords:** KIN (Kin17), cancer biomarker, protein–protein interactions, BioID-MS, splicing process, ribosome biogenesis

## Abstract

KIN (Kin17) protein is overexpressed in a number of cancerous cell lines, and is therefore considered a possible cancer biomarker. It is a well-conserved protein across eukaryotes and is ubiquitously expressed in all cell types studied, suggesting an important role in the maintenance of basic cellular function which is yet to be well determined. Early studies on KIN suggested that this nuclear protein plays a role in cellular mechanisms such as DNA replication and/or repair; however, its association with chromatin depends on its methylation state. In order to provide a better understanding of the cellular role of this protein, we investigated its interactome by proximity-dependent biotin identification coupled to mass spectrometry (BioID-MS), used for identification of protein–protein interactions. Our analyses detected interaction with a novel set of proteins and reinforced previous observations linking KIN to factors involved in RNA processing, notably pre-mRNA splicing and ribosome biogenesis. However, little evidence supports that this protein is directly coupled to DNA replication and/or repair processes, as previously suggested. Furthermore, a novel interaction was observed with PRMT7 (protein arginine methyltransferase 7) and we demonstrated that KIN is modified by this enzyme. This interactome analysis indicates that KIN is associated with several cell metabolism functions, and shows for the first time an association with ribosome biogenesis, suggesting that KIN is likely a moonlight protein.

## 1. Introduction

KIN is described as a potential diagnostic biomarker and a potential target for breast cancer therapy [1,2], as well as a suitable marker for predicting chemotherapy response in colorectal carcinoma [3]. A study recently performed and published by our group demonstrated that KIN is differently expressed in subpopulations of melanoma from murine cell lines, according to its aggressiveness [4]. Studies of KIN in human colorectal cancer cells, using siRNA to silence its expression, indicated reduced cell proliferation and an accumulation of cells in the beginning or middle of cell cycle S phase [5]. KIN is overexpressed in most cancer cells studied so far [1,6,7], except for the cell line derived from MeWo melanoma [8].

The DNA- and RNA-binding protein KIN, also known as Kin17, is well conserved among species from lower to higher eukaryotes, suggesting an important role in the maintenance of basic cellular function which remains to be defined [9]. It was identified by antibodies raised against bacterial *Escherichia coli* repair protein RecA, in an effort to identify mammalian orthologs [10]. KIN was first described as having an important function in DNA repair [11,12] and also in the initiation of DNA replication [13,14]. However, purifications of the spliceosome instead indicated an association of KIN with the ribonucleoprotein complex [15]. Immunofluorescence techniques and electronic microscopy demonstrated that KIN is associated with chromatin, but is also present in the nucleoplasm; these results are consistent with either function described [16,17].

**Figure 1:**
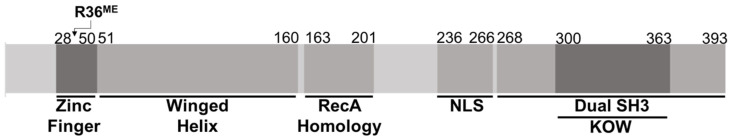
KIN protein architecture domain. Numbers indicate residues and bars indicate KIN protein domains. The up arrow indicates the arginine residue that is methylated by the PRMT7 enzyme.

KIN domain architecture (Figure 1) consists of a C2H2-type zinc finger in the N-terminal portion which has dual affinity for DNA and RNA, a winged helix domain, believed to be involved in protein–protein interactions [16] from residues 51 to 160, a purported RecA homology domain spanning residues 163–201, and a dual SH3-like domain containing a KOW motif, which is an RNA-binding module at its C-terminus [18,19,20]. The apparent multitude of identified RNA-binding domains is consistent with a role in RNA metabolism [21,22]. A recent biophysical evaluation revealed new insights into the recombinant human KIN protein, (_HSA_KIN), where the secondary structure is composed of more than 50% unfolded elements such as random coils and β-turns [9], suggesting a protein with high flexibility. Additionally, phylogenetic studies of the conserved sequences show that the KOW motif present in the KIN protein is conserved only in higher eukaryotes [9].

In the current manuscript, we present interactors of KIN identified by BioID, a proximity-dependent biotin identification methodology [23], in which a mutated biotin ligase (BirA*) is fused to our protein of interest, allowing for the local activation of biotin and subsequent biotinylation of proteins in its vicinity. We identified interactors that are components of the SSU processome and the spliceosome, implying a function for KIN in the processes of ribosome biogenesis and/or splicing. In an effort to promote conditions where interactions with proteins that are related to the processes of DNA replication and/or repair might occur, we also performed BioID at different stages of the cell cycle, as well as following irradiation; however, we found little evidence to substantiate the direct implication of KIN in DNA replication and/or repair, as previously reported. Of note, we observed that KIN interacts with the methyltransferase PRMT7 and demonstrated that it is monomethylated at arginine 36, consistent with PRMT7′s arginine methyltransferase type III activity [24]. This is the first report of such association, which was moreover confirmed by an in vitro methylation assay.

## 2. Materials and Methods

### 2.1. Cell Culture for BioID

Flp-In T-Rex 293 (Invitrogen) and Flp-In T-Rex HeLa [25] cell lines were cultured in DMEM medium (Gibco) containing 10% fetal bovine serum (Wisent), penicillin/streptomycin (100 U/mL) (Gibco), and glutamine (2mM) (Gibco). Cells were grown at 37 °C in a humidified cabinet under 5% CO_2_ to a confluence of 70–80% for transfection.

### 2.2. Generation of BirA* Fusion Protein Expression Cell Lines

Coding sequences for KIN, BUD13, and the control GFP-NLS were obtained from the National Centre for Biotechnology Information (www.ncbi.nlm.nih.gov, accessed on 2 March 2015) and primers with the recombination sites were designed (Gateway Technology, Invitrogen). PCR reactions were performed using the Q5 High Fidelity DNA Polymerase (NEB), according to the manufacturer’s recommendations. BirA*-fusion protein expression plasmids were then constructed using the Gateway Cloning System (Gateway Technology, Invitrogen) and, as final vectors, plasmids V8164 and V8449 [25]—graciously provided by A.C. Gingras (Toronto, Canada)—were used. After the sequences were confirmed by DNA sequencing, Flp-In T-Rex 293 cells (Invitrogen), or Flp-In T-Rex HeLa cells [26] were co-transfected with our final vectors and pOG44 (FLP-recombinase expression vector, Invitrogen), using the Jet Prime Transfection Kit (Polyplus, France), according to the manufacturer’s recommendations. The medium was changed 24 h after the transfection, and after an additional 24 h it was supplemented with hygromicin B (200 μg/mL) (Calbiochem). Selected cells were expanded to a 150 mm plate.

### 2.3. Induction and Harvest

Once cells were subconfluent (60–70%), they were induced with tetracycline 1 μg/mL (Bioshop) and Biotin 75 μM (BioBasic) was added for biotinylation. Cells were harvested 24 h after being induced.

### 2.4. Synchronization

Stable Flp-In T-Rex HeLa cells were synchronized according to methods previously described in [26,27]. Briefly, cells were treated with a double thymidine block procedure, which involves 2 mM thymidine in complete medium for 16 h, release into the cell cycle for eight hours by washing out thymidine, and then 2 mM thymidine in complete medium for 16 h again. The block was released and cells were harvested at 0, 6, and 12 h; one replicate was analyzed by FACS to confirm the cell cycle stage, while the other was induced for BioID purification. For FACS analysis, DNA staining was performed using propidium iodide according to the modified Krishan procedure [28]. Cells were sorted using FACScan (BD Bioscience) and the data analyzed with Modfit LT (Figure S1).

### 2.5. Irradiation

Stable cell lines, expressing KIN or GFP-NLS, were irradiated using gamma rays at 10 grays and harvested 2 or 24 h later for BioID purification; non-irradiated cells were used as control. For DNA damage identification, cells were cultivated on coverslips at the bottom of the plate, and underwent the same irradiation treatment. Cells were fixed in freshly prepared 4% paraformaldehyde in PBS, permeabilized in 0.5% Triton X-100 in PBS, blocked in 2.5% goat serum, 1% BSA. Cells were incubated in anti-H2AX (monoclonal #05-636, Millipore) 1:5000, and goat-anti-mouse Alexa Fluor 546 (Thermo Fisher Scientific, Waltham, MA, USA) 1:500. Nuclear staining was performed using DAPI (Sigma, Burlington, VT, USA), and coverslips were mounted on slides using one drop of mounting medium (Aqua-Mount #13800, Thermo Fisher Scientific). Images were acquired using the DM4000 fluorescent microscope (Leica, Wetzlar, Germany). One replicate of KIN and one replicate of GFP-NLS were used for each point, non-irradiated, irradiated and harvested after two hours, and irradiated and harvested 24 h later (Figure S2).

### 2.6. BioID Purification

BioID purification was performed according to Coyaud et al. [29] with modifications. Briefly, 0.1 g of cells were harvested and washed in cold PBS 1X, then lysed in 2 mL modified RIPA lysis buffer (50 mM Tris-HCL, pH 7.5, 150 mM NaCl, 1 mM EDTA, 1 mM EGTA, 1% Triton X-100, 0.1% SDS, 1X protease inhibitor cocktail—Sigma Fast 10X) before 1 μL of Benzonase 250 U (Novagen) was added prior to sonication (30 sec at 30% amplitude). The lysate was centrifuged for 30 min at 12,000 rpm and the clarified supernatant was incubated with 35 μL of pre-equilibrated streptavidin-agarose beads (GE) at 4 °C for 3 h with rotation. An aliquot of the clarified supernatant was saved for western blotting analysis. Beads were collected by centrifugation (2 min at 2000 rpm), washed twice in modified RIPA lysis buffer, three times in 50 mM ammonium bicarbonate pH 8.5 (ABC), and resuspended in 100 μL of ABC for trypsin digestion.

### 2.7. Digestion and LC-MS/MS

Trypsin digestion and LC-MS/MS were performed according to the method previously described in [30]. For BioID, it was recommended to release the peptides for analysis by MS using on-bead tryptic digestion, which also prevents removal of the streptavidin itself from the beads that might interfere with MS analysis [31], therefore, the on-bead proteins were digested with 0.5 μg sequencing grade modified trypsin (Promega, Madison, WI, USA) overnight at 37 °C with agitation. The supernatants were collected and the beads were washed two times with 100 μL water. The supernatants of each wash were pooled and then reduced and alkylated. The reduction step was performed with 9 mM dithiothreitol at 37 °C and, after cooling for 10 min, the alkylation step was performed with 17 mM iodoacetamide at room temperature for 20 min in the dark. The supernatants were acidified with trifluoroacetic acid for desalting and removal of residual detergents by MCX (Waters Oasis MCX 96-well Elution Plate) following the manufacturer’s instructions. After elution in 10% ammonium hydroxide/90% methanol (v/v), samples were concentrated with a speed-vac and reconstituted in 2% acetonitrile, 1% formic acid. Desalted tryptic peptides were loaded onto a 75  μm i.d. × 150 mm Self-Pack C18 column installed in the Easy-nLC II system (Proxeon Biosystems, Roskilde, Denmark). The buffers used for chromatography were 0.2% formic acid (buffer A) and 100% acetonitrile/0.2% formic acid (buffer B). Peptides were eluted with a two-slope gradient at a flowrate of 250 nL.min^−1^. Solvent B first increased from 2 to 40% in 82 min and then from 40 to 80% B in 28 min. The HPLC system was coupled to a LTQ Orbitrap Velos mass spectrometer (Thermo Scientific) through a nano-ESI source (Proxeon Biosystems, Roskilde, Denmark). Nanospray and S-lens voltages were set to 1.3–1.8 kV and 50 V, respectively. Capillary temperature was set to 225 °C. Full scan MS survey spectra (*m*/*z* 1360–2000) in profile mode were acquired in the Orbitrap with a resolution of 60,000 with a target value at 1e6. The ten most intense peptide ions were fragmented by collision induced dissociation in the LTQ with a target value at 1e4 (normalized collision energy 35 V, activation Q 0.25, and activation time 10 ms). Target ions selected for fragmentation were dynamically excluded for 25 s. The peak list files were generated with Proteome Discoverer (version 2.1) using the following parameters: minimum mass set to 500 Da, maximum mass set to 6 kDa, no grouping of MS/MS spectra, precursor charge set to auto, and minimum number of fragment ions set to 5.

### 2.8. Computational Analyses

Data analysis was performed as described by Cloutier and collaborators [32]. Protein database searching was performed with Mascot 2.3 (Matrix Science, Columbia, CA, USA) against the human NCBInr protein database (version 18 July 2012). The mass tolerances for precursor and fragment ions were set to 10 ppm and 0.6 Da, respectively. Trypsin was used as the enzyme allowing for up to one missed cleavage. Cysteine carbamidomethylation was specified as a fixed modification, and methionine oxidation as variable modifications. In cases where multiple gene products were identified from the same peptide set, all were unambiguously removed from the data set. When multiple isoforms were identified for a unique gene, only the isoform with the best sequence coverage was reported. Proteins identified on the basis of a single spectrum were also discarded. Reliability of the data obtained was assessed using SAINTexpress version 3.6.1 [33,34], which assigns a false discovery rate (FDR) to each protein–protein interactions. The threshold FDR score was set at 0.1 (Table S1).

### 2.9. Interactome Design

For KIN, we worked with three processual and two biological replicates, and true interactors were determined when the protein was detected in all of the four replicates and either not present or 50 percent more spectrum counts, compared to the control. For BUD13, we worked with two biological replicates, and true interactors were defined the same way. For each replicate we used a GFP-NLS replicate as control. The interactome was designed using the program Cytoscape (version 3.0, downloaded from http://www.cytoscape.org, accessed on 10 February 2016) [35] (Table S2).

### 2.10. Immunolocalization

HeLa cells were grown on coverslips to a 30–40% confluency in RPMI medium supplemented with FBS 10%, glutamine 1%, and streptomycin/penicillin 1:100, at 37 °C in 5% CO_2_. Cells were fixed with 4% paraformaldehyde for 10 min, permeabilized by 0.5% Triton X-100 for 10 min, and blocked with PBS containing 3% BSA and 20% goat serum (Sigma) for one hour. The primary antibody anti-kin17 K58 (sc-32769; Santa Cruz Biotechnology) was diluted 1:500 in blocking solution and then added to the cells for 60 min. Alexa Fluor 350-conjugated goat anti-mouse secondary antibody (A11045; Molecular Probes) was diluted 1:4000 and incubated for 60 min. As a nucleolar marker, Anti-Fibrillarin antibody (Abcam AB5821) was used 1:500, incubated for 60 min, and detected by Alexa Fluor 488-conjugated donkey anti-rabbit secondary antibody (A21206; Molecular Probes), which was diluted 1:4000, incubated for one hour. Nuclear chromatin was stained with propidium iodide (4 mg/mL) for 5 min. All staining reactions took place at 37 °C. Images were obtained using an Olympus FSX-100 microscope.

### 2.11. In Vitro Methylation Assay

The in vitro methylation assay was performed according to methods previously described in [36]. The coding sequence of KIN was cloned in pET-23a(+) vector (EMD Chemicals, Burlington, VT, USA), and methyltransferases were cloned in to pGEX-4-T1 vector (GE Healthcare, Chicago, IL, USA). Vectors were transformed in One Shot BL21 Star (DE3) (Life Technologies) and protein synthesis was induced with IPTG. Bacteria were harvested by centrifugation and pellets were lysed with the use of an IEC French Press (Thermo Scientific). The resulting proteins were purified using Ni-NTA Agarose beads (QIAGEN, Hilden, Germany), and Glutathione Sepharose 4B (GE Healthcare, Chicago, IL, USA), according to the manufacture’s recommendations. For each reaction, 1 μg of GST-tagged methyltransferase was incubated with 2.5 μg His-tagged KIN and 5 μCi of S-(methyl-^3^H)-Adenosyl-L-methionine (81.7 Ci/mmol; PerkinElmer, Norwalk, CT, USA), according to the manufacture’s specifications. The samples were resolved in a 10% acrylamide gel which was then treated with EN^3^HANCE (PerkinElmer), according to the manufacturer’s recommendations. Tritium-based methylation signals were detected by radiography after 24 h of exposure on Amersham Hyperfilm MP (GE Healthcare, Chicago, IL, USA) at −80 °C.

## 3. Results

### 3.1. KIN Mostly Associates with Proteins Involved in Ribosome Biogenesis and Splicing

**Figure 2:**
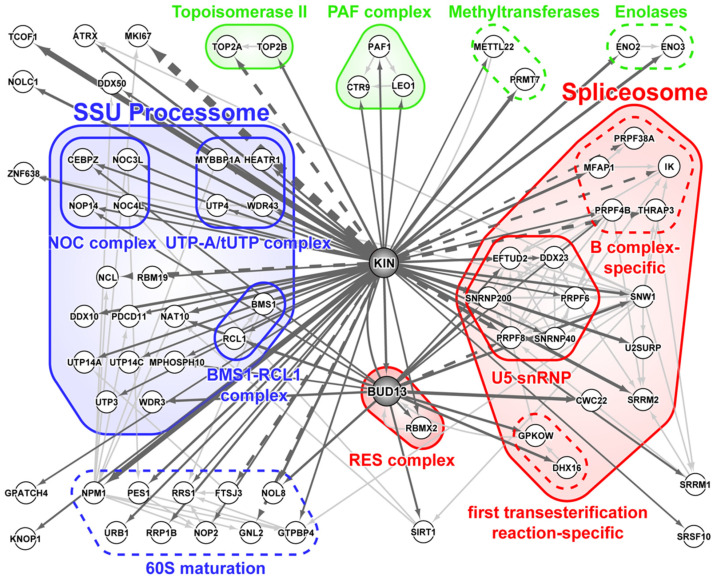
Protein–protein interaction network of KIN and BUD13. Proteins purified by BioID/MS. Interaction map designed using Cytoscape 3.0. Thickness of arrows are according to number of spectral counts. Full lines indicate a FDR of zero and broken lines indicate FDR greater than zero. Proteins were grouped according to their functions.

Cellular processes depend on protein interactions, and in order to better understand KIN function, we defined its interaction network (Figure 2) based on the BioID experiments where recombinant KIN proteins were expressed with BirA* at either its N- or C-terminus, thereby allowing the identification of interactors whose binding might have been lost due to interference by the positioning of the modified biotin ligase. Most of its interactors are associated with the processes of ribosome biogenesis and splicing. We also identified topoisomerases TOP2A and TOP2B and enolases ENO2 and ENO3—members of the PAF complex, which is known to interact with RNA polymerase II and may have a post-transcriptional role in mRNA processing and maturation [37]. Methyltransferase PRMT7 is reported and confimed, as an interactor for the first time in the present article; moreover, we confirmed the interaction with methyltransferase METTL22, which was previously reported by Cloutier and collaborators [36,38].

The small-subunit (SSU) processome is a large macromolecular complex, also termed 90S pre-ribosome, that is formed in the nucleolus by assembly factors and small nucleolar RNA U3 during the early stages of ribosomal biogenesis [39,40]. KIN interacts with many proteins of the SSU processome, notably components of the NOC, UTP-A/tUTP, and BMS1-RCL1 subcomplexes, as well as proteins that are involved in subsequent maturation of the 60S ribosomal subunit.

BioID of KIN also identified a number of splicing factors and components of the spliceosome, a highly complex and dynamic ribonucleoparticle comprising five non-coding small nuclear RNAs (U1, U2, U4, U5, and U6 snRNAs) and more than 200 proteins associated at one or more stages of the splicing cycle [41]. In essence, 5’ and 3’ intron boundaries are first recognized by U1 snRNP splicing factor 1 (SF1) and U2 auxiliary factor (U2AF). This state is known as complex E and is followed by the arrival of U2 snRNP, bringing about the complex A form of the pre-spliceosome. At this point, the rest of the snRNPs (U4/U6, U5 tri-snRNP) are integrated into the growing complex to form a pre-catalytic spliceosome known as complex B. Extensive snRNA interaction and protein composition rearrangements then take place, resulting in the intermediary complex B* followed by the catalytic complex C, the latter of which is poised to perform the two sequential transesterification reactions that will remove the intron from the pre-mRNA and ligate the two flanking exons together. The remaining spliceosomal components still present on the mRNA are known as the post-spliceosome or complex C*. Interestingly, identified spliceosome subunits in the KIN BioID experiment mostly belong to the complex B stage, in particular the B complex-specific proteins PRPF38A, PRPF4B, MFAP1, THRAP3, and IK (Figure 2). KIN, likewise, showed significant affinity toward lesser known retention and splicing complex (RES), a heterotrimeric complex studied primarily in *Saccharomyces cerevisiae* and composed of BUD13, RBMX2/Snu17, and SNIP1/Pml1 [42,43]. Association of KIN with BUD13 has already been noted in multiple studies [36,44], and represents its highest confidence interactor. For this reason, we elected to perform BioID on BUD13 as well (Figure 2). Strikingly, the SNIP1 subunit of the RES complex was never observed in either KIN or BUD13 BioID experiments. KIN and BUD13 did show similarity in their affinity towards both the ribosomal biogenesis machinery and spliceosome, although the identified components only showed partial overlap.

### 3.2. DNA Replication and/or Repair Proteins Were Not Obvious Interactors in Synchronized and/or Irradiated Cells

**Figure 3:**
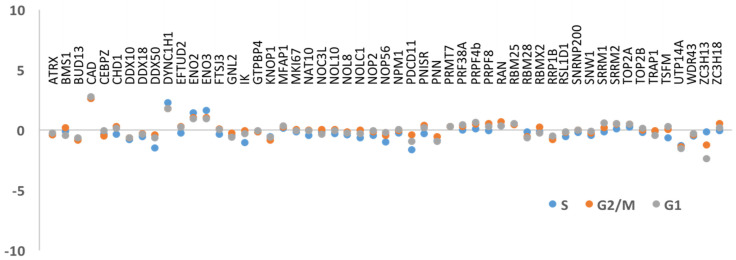
Protein-protein interaction in synchronized cells. The results show the lack of variation of interactors in the S and G2/M phases of the synchronized cells.

**Figure 4:**
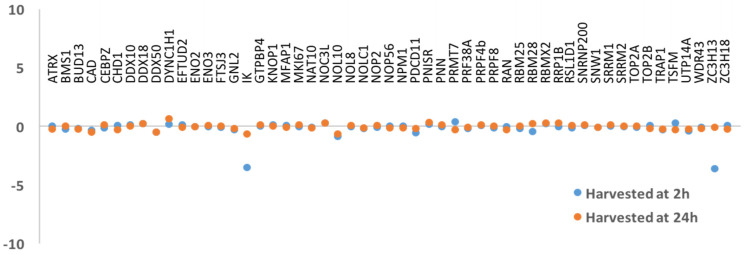
Protein-protein interaction in irradiated cells. The results show the lack of variation between interactors at 2 and 24 h after the irradiation experiment.

Despite the published data reporting KIN as a protein involved in DNA repair and replication, our results could not substantiate these functions as there were few proteins involved in either of these processes in any of our purifications. However, replication and DNA repair are transient cellular states that were likely underrepresented in the normal growing conditions used to build our KIN interactome. In order to promote identification of KIN interactors involved in replication, we synchronized cells at different stages of the cell cycle (Figure S1). We compared the interactors found in non-synchronized cells against interactors in cells in different phases of the cell cycle (S, G2/M, and G1), and the results demonstrated that no major variation was detected amongst the S and G2/M stages (Figure 3). We did not observe a significant enrichment in any of the previously reported interactions, such as RPA1, RPA2, PCNA, or DNA polymerase α [13,14]. Although we were able to identify interactors that are known to participate in the DNA replication and/or transcription processes, such as topoisomerases (TOP2A and TOP2B), as well as ATRX, CEBZ, and NOC3L [18,45], we did not observe any significant changes in affinity in any of the stages of the cell cycle studied in this experiment. Next, to promote identification of KIN interactors involved in DNA repair, we subjected cells to gamma irradiation, which causes DNA breakage and induces DNA repair. BioID was performed as before, and interactors present 2 h after irradiation as well as 24 h later were examined. Again, we could not detect significant variation amongst these proteins (Figure 4), nor could we identify additional ones.

### 3.3. KIN Is Present in the Nucleolus as Well as Other Parts of the Nucleus

**Figure 5:**
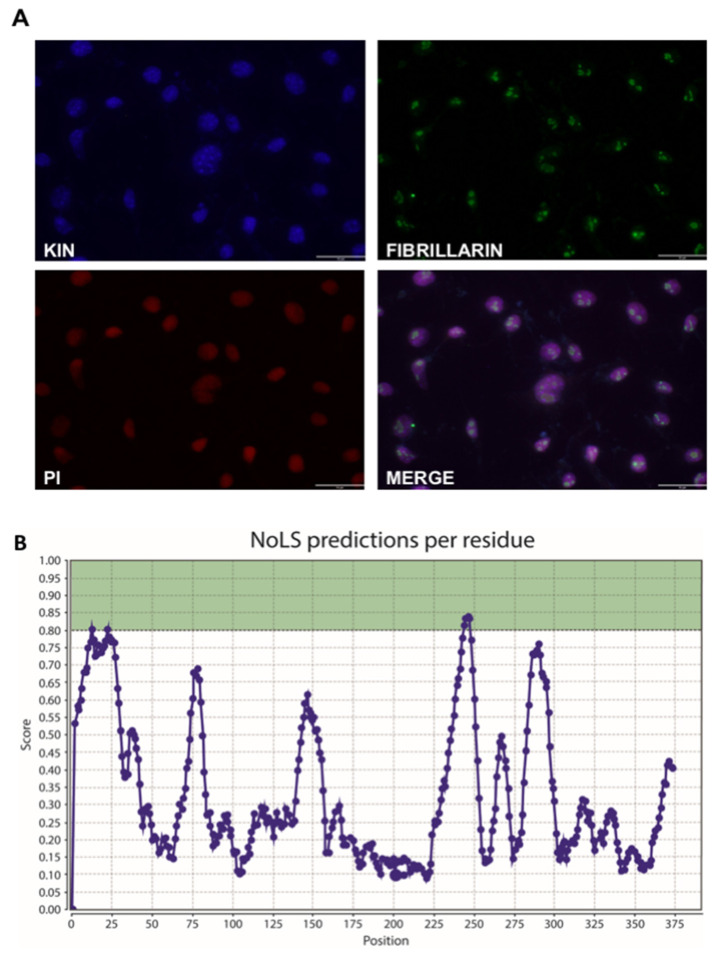
KIN protein immunolocalization. (**A**) Immunofluorescence of HeLa cells shows KIN present at the nucleolus, as well as in other parts of the nucleus. Scale bar: 32 µm. (**B**) Predicted nucleolar localization signal by NOD.

Our identification of numerous ribosome biogenesis factors associated with KIN is somewhat at odds with a previous report of the nuclear localization of the protein, which noted its absence from the nucleolus, where ribosome maturation takes place [11]. We therefore performed our own immunolocalization experiments which showed uniform nuclear localization, including within nucleoli (Figure 5A). This result is strengthened by previous identification of KIN as a component of the nucleolus [46], and observation of its accumulation in nucleoli of tobacco leaf epidermal cells [47]. Localization of KIN in the nucleoli, as well as in other parts of the nucleus, supports the results proposed here, where proteins related to pre-mRNA splicing and ribosomal biogenesis processes represent the majority of proteins detected in this analysis. However, no nucleolar signal has been identified in KIN to date. Using NoD (Nucleolar Localization Signal Detector, (http://www.compbio.dundee.ac.uk/www-nod/index.jsp, accessed on 20 May 2016) [48], we were able to discern two putative NoLS (Figure 5B). The first one, between positions 12 and 41, falls within a highly structured domain corresponding to the zinc finger and is, in all likelihood, not a functional NoLS. The second site, however, between positions 244 and 266 (SSQSSTQSKEKKKKKSALDEIME), overlaps with the previously identified nuclear localization signal (NLS). Both features are typically similar due to their acidic residue content, thus overlap or dual function is often observed in many nucleolar proteins.

### 3.4. Methyltransferase PRMT7 Methylates KIN at Arginine 36

**Figure 6:**
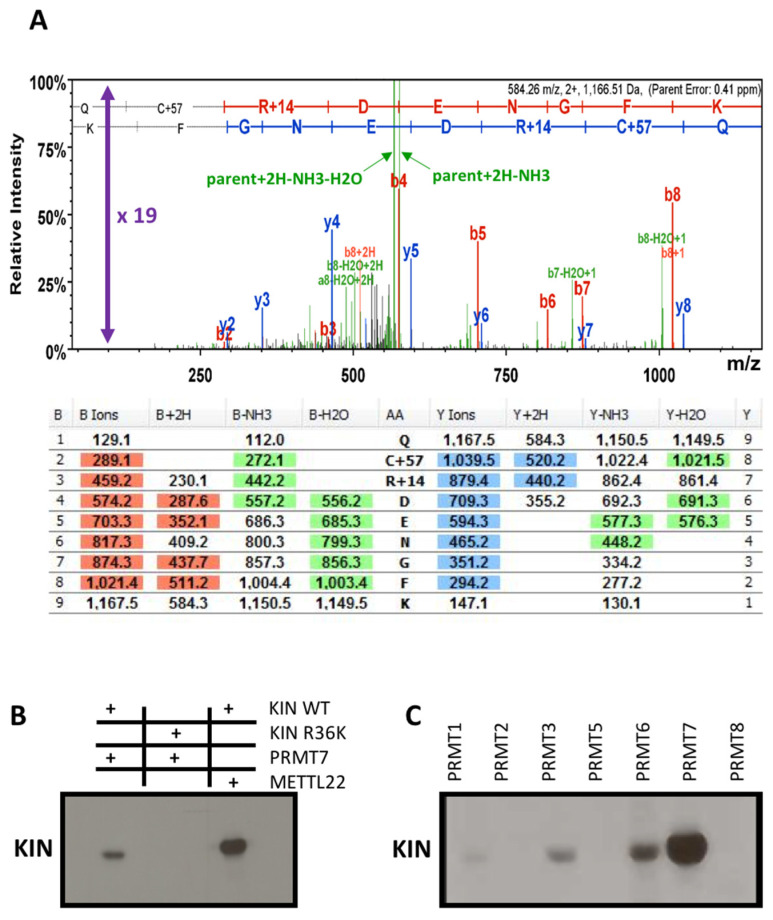
KIN protein methylation assay. (**A**) Monomethylation at arginine on position 36; (**B**) In vitro methylation assay with tritium-labeled S-(methyl-^3^H)-Adenosyl-L-methionine, PRMT7 enzyme, and KIN-His wild type and KIN-His mutated R36K. (**C**)**.** In vitro methylation assay with tritium-labeled S-(methyl-^3^H)-Adenosyl-L-methionine, GST-PRMT family of proteins, and KIN-His. The protein KIN demonstrated strongest affinity with PRMT7.

Methylation is a common post-translational modification, where the addition of one or more methyl groups to a protein can ultimately impact its cellular localization, turnover, activity, or molecular interactions. Methyltransferases are a group of proteins that transfer a methyl group from a donor molecule, usually an S-adenosylmethionine, to the target protein. Side chains of arginine and lysine residues are the most commonly methylated residue substrates [38]. It was previously reported that methyltransferase METTL22 trimethylates KIN at residue K135, affecting its cellular localization and association with chromatin [36]. In hopes of identifying additional regulatory post-translational modifications, we queried the data from our experiments for a number of possible PTMs and observed monomethylation of KIN at arginine 36 (Figure 6A). Interestingly, PRMT7, the novel methyltransferase identified in our BioID experiments as a novel interactor of KIN, belongs to a family of arginine methyltransferases. However, while most members enact symmetric or asymmetric dimethylation (type II and type I activity, respectively), PRMT7 is the only one among them that can specifically perform monomethylation (type III activity) [24,49]. An in vitro methylation assay was performed to confirm methylation of KIN by PRMT7. Methylation of wild-type KIN by PRMT7 was indeed observed with a robust intensity that almost rivals that of previously reported methylation by METTL22. However, when arginine 36 is substituted by a lysine, the signal is lost, confirming that PRMT7 indeed performs methylation at this site (Figure 6B). Methyltransferase domains of PRMTs are highly homologous and partial overlap in specificity is often noted. In light of this, various PRMT family members were tested for their ability to methylate KIN. Although PRMT1, PRMT3, and PRMT6 were able to weakly modify the protein, the signal was significantly stronger in the presence of PRMT7 (Figure 6C).

## 4. Discussion

Understanding the interaction network of a protein is an important tool in characterizing its function within a cell’s metabolism. Transient interactions are quite difficult to detect by traditional affinity purification methodologies as they are performed with cell lysate. The presented results, acquired by biotinylation performed in live cells, allows for the advantageous identification of proteins that do not necessarily form stable complexes and thus the detection of transient interactors. Kim and collaborators [50] showed that not only intra-complexes but also inter-complexes proteins can be identified by this technique in a radius of approximately 10–20 nm; moreover, it also identified interactors that might be less abundant [51]. Our purification detected a greater number of proteins which, however, belong to the same processes of ribosome biogenesis and/or splicing as previously reported by tandem affinity purification (TAP) [36]. The additional information that we present with our results is as follows: whereas TAP mostly identified ribosomal proteins of the 60S ribosomal subunit, BioID revealed complexes associated with earlier steps in ribogenesis, notably the SSU processome and 60S-specific maturation factors, all of which act prior to the inclusion of ribosomal proteins to form the final RNP complex. In this case, the different affinity purification techniques proved to be complementary in identifying the pathway where KIN may play a role.

Association with the spliceosome was also observed by TAP purification [36], although the identified proteins were limited to some of the largest subunits of the U5 snRNP. This is likely due to the low abundance of co-purified spliceosome. Indeed, large proteins are more readily detected by mass spectrometry as they generate more tryptic peptides. The spliceosome is by no means a low-abundance complex, but by virtue of its dynamic nature, a protein could be transiently associated with a single step of the splicing cycle only to be released afterwards, making co-purification by traditional AP-MS techniques challenging. This seems to be the case as BioID of KIN identified a number of factors specific to the pre-catalytic B complex form of the spliceosome, consistent with what was observed by Hegele et al. [41]. Interestingly, BioID of KIN interactor BUD13 also demonstrated association with both the spliceosome and ribosomal maturation complexes. BioID of Bud13 identified a number of factors specific to the activated B* complex form of the spliceosome (CWC22) (Figure 2), as well as factors associated with the first transesterification reaction of splicing (GPKOW and DHX16). It should be noted that GPKOW is yet another KOW motif-containing protein, raising the possibility that BUD13 may play a role in substituting one KOW protein for another, sitting either on the pre-mRNA or an snRNA, to promote progression of the splicing cycle. BUD13 was likewise associated with a few ribosomal biogenesis factors distinct to the ones observed in BioID of KIN. While both proteins showed affinity towards components of the 90S pre-ribosome, KIN interacted with earlier components of the SSU processome, notably the UTP-A (also known as transcriptional UTP or tUTP), while BioID of BUD13 recovered significant amounts of WDR3, a subunit of the UTP-B subcomplex. It remains to be determined whether BUD13 can promote progression of ribogenesis by substituting KIN for another KOW protein on pre-rRNA (or U3 snoRNA), but it should be noted that a number of protein components of the mature ribosome contain this moiety (RPL6, RPL14, RPL26, RPL27, and RPS4), as well exosome cofactor MTR4, involved in rRNA processing [52].

Ribosome biogenesis is a well-coordinated, central process in cellular metabolism, crucial to cell proliferation. Mutations in genes encoding ribosomal proteins (RPs) or ribosome assembly factors are associated with cancers derived from ribosomopathies; drugs that inhibit ribosome biogenesis could thus be a viable therapeutic approach to cancer treatment [53]. This process takes place in the nucleolus, which has a well-conserved eukaryotic ultra-structure; however, the size and number of nucleoli vary according to cell type and metabolic state [52]. The nucleolus is considered to be a target for cancer intervention [54].

Analysis of the KIN architectural structure favors RNA interaction, being favorable for mRNA processing given the domains for RNA binding, namely the zinc finger in the N-terminus portion and the dual SH3-like domain at the C-terminus portion (Figure 1), confirming that KIN plays a role in mRNA processing as previously reported [15,36,38]. The most singular feature of KIN that links it to the spliceosome, ribosome, and PAF complex is its KOW motif, an RNA-binding structural element shared by splicing factor GPKOW; ribosomal proteins RPL6, RPL14, RPL26, RPL27, and RPS4; SUPT5H (DSIF subunit and interactor of the PAF complex); and TRAMP subunit MTR4, which has a connection between RNA decay and the spliceosome [55].

Unlike the RAD51 family of proteins, a well-characterized family of proteins involved in DNA repair, KIN shares very little homology with RecA; moreover, we did not purify many interactors that are part of DNA repair mechanisms, even following irradiation of the cells, causing DNA breakage, and analysis of the interactors of KIN 2 or 24 h later. We also did not purify a sufficient amount of interactors that are part of the replication process in order to confirm that KIN could be directly involved in either the replication and/or repair process. However, a link between DNA damage, response, and splicing has been widely demonstrated, as reviewed by Shkreta and Chabot [56], and there is evidence of proteins that act in both ribosome biogenesis and DNA damage such as NPM1/NCL, as reviewed by Scott and Oeffinger [57]. The interactor NCL is a multifunction protein with roles in chromatin structure and ribosome biogenesis and is one of the most abundant non-ribosomal proteins of the nucleolus. It is also found in the nucleus, cytoplasm, and cell membrane [58].

Other proteins such as PPAN, RPS3, GNL3, and NOL12 also play roles in DNA damage repair and other aspects of RNA metabolism. NOL12, for example, is an RNA-binding protein (RBP) that is involved in DNA damage response (DDR) [57]. There is increasing evidence of cross-talk between DNA repair enzymes and proteins involved in RNA metabolism that seems reasonable given that the nucleolus is emerging as a dynamic functional hub that coordinates cell growth arrest and DNA repair mechanisms [59]. KIN was recently identified as being involved in class switch recombination (CSR) for the repair of DNA double strand breaks (DSBs) [12], therefore we believe that it is indirectly involved in such a process. Several proteins were reported to play roles in ribosome biogenesis as well as splicing, with SNU13, PRP43P, and CD8 (p32) representing a few examples [60,61,62,63,64]. Multidomain proteins can interact with proteins as well as nucleic acids, and can act as adaptors to directly or indirectly participate in the interaction of proteins and/or nucleic acids, making it possible that they are present in multicompartment processes that are not related, such as replication, DNA repair, ribosomal biogenesis, and splicing.

Protein methylation is not as prevalent as in other PTMs such as phosphorylation, acetylation, or ubiquitination, and is usually limited to functionally related proteins including splicing factors and ribosomal proteins. KIN is methylated on lysine 135 by METL22 [36], and this modification was shown to affect its localization. We demonstrate that KIN is methylated by PRMT7 in arginine 36, which is within the zinc-finger domain, a nucleic acid-binding domain with diverse functions [65]. Arginine methylation, by the PRMT family, generates modifications that play roles in a multitude of regulatory pathways, with RNA-binding proteins representing a major target for such proteins [66,67], giving even more weight to the hypothesis that KIN is a multifunction protein with roles in ribosome biogenesis and/or splicing. 

Regardless of our efforts to promote conditions where interactions with proteins that are related to the processes of DNA replication and/or repair would occur, we found little evidence to substantiate the direct implication of KIN in DNA replication and/or repair, as had been previously reported in earlier studies with this protein [5,11,12,13,14]. A deeper understanding of the roles of KIN through functional studies will prove helpful for upcoming research in this area.

## Data Availability

The raw mass spectrometric data have been deposited to the ProteomeXchange Consortium via the PRIDE [35] partner repository with the dataset identifier PXD010487 and 10.6019/PXD010487.

## Supplementary Materials

The following are available online at https://www.mdpi.com/article/10.3390/cimb43020056/s1. Figure S1: Cell synchronization, Figure S2: Gamma-irradiated cells, Table S1: Proteins identified on the basis of a single spectrum (SAINTexpress version 3.6.1), Table S2: Protein interactome (AgileProteinInteractomesDataServer–APID).
