# Supplementary material for: Interactome Analysis of KIN (Kin17) Shows New Functions of This Protein
Source: Curr Issues Mol Biol. 2021 Jul 22;43(2):767–81. doi: 10.3390/cimb43020056 (PMC8929021; doi:10.3390/cimb43020056)
Supplement: Supplementary file 1 [file cimb-43-00056-s001.zip › SUPPLEMENTARY FIGURES S1 AND S2.pdf]

SUPPLEMENTARY MATERIAL

Interactome analysis of KIN (kin17) shows new functions to this protein.

Vanessa Pinatto Gaspar[1,2], Anelise Cardoso Ramos[1], Philippe Cloutier[2], José Renato Pattaro Junior[3], Francisco Ferreira Duarte Junior[1], Annie Bouchard[2], Flavio Augusto Vicente Seixas[3], Benoit Coulombe[2], Maria Aparecida Fernandez[1*]

[1]Departamento de Biotecnologia, Genética e Biologia Celular. Universidade Estadual de Maringá, Av. Colombo, 5790, 87020-900, Maringá, Paraná – Brazil. vapigas@gmail.com, anelise.andre@gmail.com, junior.fduarte@gmail.com, mafernandez@uem.br
[2]Institut de Recherches Cliniques de Montréal, 110 Avenue des Pins Ouest - H2W 1R7, Montreal, QC, Canada. phil.clou@gmail.com, anniebouchard78@gmail.com, benoit.coulombe@ircm.qc.ca
[3]Departamento de Tecnologia, Universidade Estadual de Maringá, campus Umuarama, Av. Ângelo Moreira da Fonseca, 1800, 87506-370 Umuarama-Paraná, Brazil; pattoze@gmail.com, favseixas@gmail.com
[1*] **Corresponding author:** Maria Aparecida Fernandez, Universidade Estadual de Maringá, Bloco B36 - Av. Colombo, 5790, 87020-900, Maringá, Paraná – Brazil. +554430115398; mafernandez@uem.br; aparecidafernandez@gmail.com

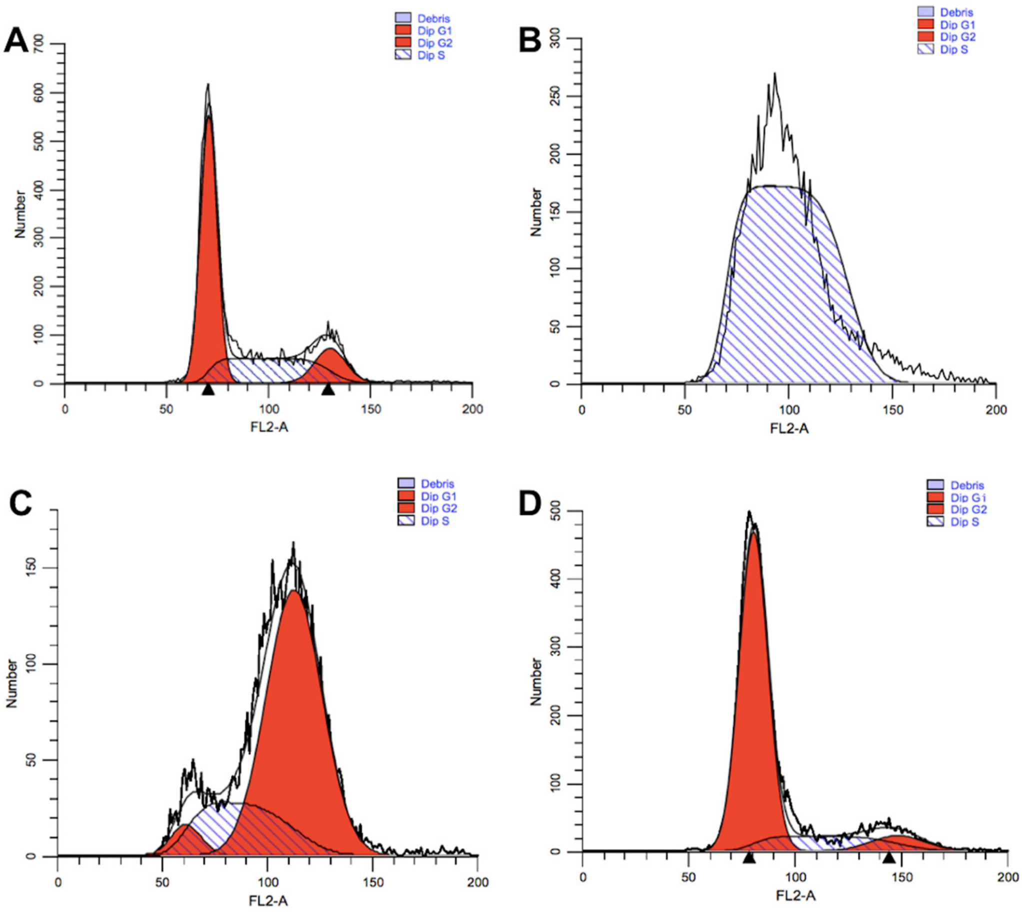

Figure S1. Cell Synchronization. **A.** Non-synchronized cells; **B.** 0h after thymidine block release; **C.** 6 hours after thymidine block release; **D.** 12 hours after thymidine block release.

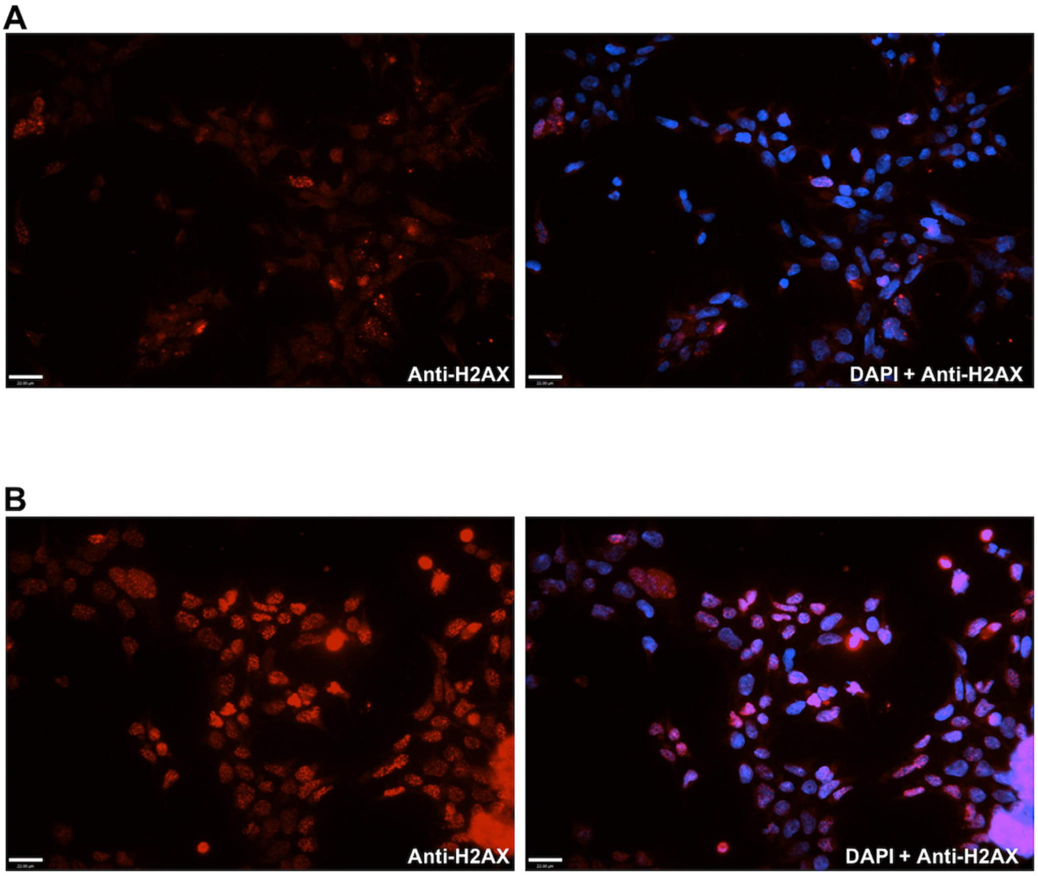

Figure S2. Gamma-irradiated cells. Anti-H2AX shows DNA damage. **A.** Non-irradiated cells; **B.** Irradiated cells. Scale bar: 32μm.
